# Development of a risk score for predicting the benefit versus harm of extending dual antiplatelet therapy beyond 6 months following percutaneous coronary intervention for stable coronary artery disease

**DOI:** 10.1371/journal.pone.0209661

**Published:** 2019-02-14

**Authors:** Guy Witberg, Ygal Plakht, Tamir Bental, Becca S. Feldman, Maya Leventer-Roberts, Amos Levi, Hagit Gabay, Ran Balicer, Yariv Gerber, Ran Kornowski

**Affiliations:** 1 The Department of cardiology, Rabin Medical Center, Petah-Tikva, Israel; 2 The Sackler school of medicine, Tel-Aviv University, Tel-Aviv, Israel; 3 Department of Epidemiology and Preventive Medicine, School of Public Health, Sackler Faculty of Medicine, Tel Aviv University, Tel Aviv, Israel; 4 Clalit Research Institute, Chief Physician’s Office, Clalit health Services, Tel-Aviv, Israel; 5 Faculty of Health Sciences, Ben-Gurion University of the Negev, Beer-Sheeva, Israel; 6 Icahn School of Medicine at Mount Sinai, New York, New York, United States of America; San Giovanni Addolorata Hospital, ITALY

## Abstract

**Background:**

Decisions on dual antiplatelet therapy (DAPT) duration should balance the opposing risks of ischaemia and bleeding. Our aim was to develop a risk score to identify stable coronary artery disease (SCAD) patients undergoing PCI who would benefit or suffer from extending DAPT beyond 6 months.

**Methods:**

Retrospective analysis of a cohort of patients who completed 6 months of DAPT following PCI. Predictors of ischaemic and bleeding events for the 6–12 month period post-PCI were identified and a risk score was developed to estimate the likelihood of benefiting from extending DAPT beyond 6 months. Incidence of mortality, ischaemic and bleeding events for patients treated with DAPT for 6 vs. 6–12 months, was compared, stratified by strata of the risk score.

**Results:**

The study included 2,699 patients. Over 6 months’ follow up, there were 78 (2.9%) ischaemic and 43 (1.6%) bleeding events. Four variables (heart failure, left ventricular ejection fraction ≤30%, left main or three vessel CAD, status post (s/p) PCI and s/p stroke) predicted ischemic events, two variables (age>75, haemoglobin <10 g/dL) predicted bleeding. In the lower stratum of the risk score, 6–12 months of treatment with DAPT resulted in increased bleeding (p = 0.045) with no decrease in ischaemic events. In the upper stratum, 6–12 months DAPT was associated with reduced ischaemic events (p = 0.029), with no increase in bleeding.

**Conclusion:**

In a population of SCAD patients who completed 6 months of DAPT, a risk score for subsequent ischaemic and bleeding events identified patients likely to benefit from continuing or stopping DAPT.

## Introduction

The optimal duration of dual antiplatelet treatment (DAPT) in patients undergoing percutaneous coronary intervention (PCI) is one of the most debated issues in interventional cardiology practice. The most comprehensive review of clinical data suggested that as the DAPT duration is extended, there is a trade-off between reduced ischemic events (stent thrombosis and myocardial infarction) and increased systemic bleeding risk, with a neutral effect on mortality [1]. The current American and European practice guidelines recommend a DAPT duration according to the clinical presentation, e.g. 6 months for patients undergoing PCI due to stable coronary artery disease (SCAD) and 12 months in cases of acute coronary syndromes (ACS). The guidelines stress the need to customise the duration of DAPT according to the relative risks for ischaemia versus bleeding [2, 3]. Unfortunately, evaluating and balancing these risks is challenging using clinical judgment alone [4]. Over recent years, validated risk assessment tools have been developed to add a more objective component to this assessment. The DAPT [5] and PRECISE-DAPT [6] scores were published and integrated into the above-mentioned guidelines [3]. Both scores have significant limitations when applied to patients who undergo PCI due to SCAD, who constitute half of the PCI population [7]. The DAPT score was designed to predict the overall benefit versus harm of extending DAPT beyond 12 months while for SCAD patients, the decision on DAPT cessation or prolongation needs to be addressed at 6 months post-PCI. In addition, both scores are based on derivation cohorts with a predominance of ACS patients and on patient populations that were enrolled in randomised clinical trials; hence, their generalizability for the overall PCI population is uncertain. Indeed, as recently reported, the DAPT score performed poorly when applied to the participants of the ISAR-SAFE trial, in which 60% of participants had SCAD or silent ischemia [8,9], and the PRODIGY trial, in which only 26% of participants were SCAD patients [10,11]. This study addressed these knowledge gaps using a large registry of patients undergoing PCI due to SCAD. Our goals were to: 1) identify baseline characteristics associated with reduced ischaemic events and/or increased bleeding events from extending DAPT duration beyond 6 months, 2) develop a risk score, and (3) assess the effectiveness of that risk score in identifying those patients most likely to benefit/suffer from extending DAPT beyond 6 months.

## Materials and methods

This was a retrospective study based on a large PCI registry. The data source included SCAD patients undergoing PCI who were identified from the Rabin Medical Centre (RMC) registry that contains detailed demographic, clinical, and angiographic data on all cases of PCI at a tertiary, academic medical centre from January 1^st^ 2004 [12]. The patients were than identified using their national identification number in the Clalit health Services (CHS) Services, the largest health management organization within the Israeli national healthcare system. CHS maintains a complete and comprehensive clinical and administrative data warehouse that includes lab results, medications prescribed and procured, as well as the International Classification of Diseases, Ninth Revision, Clinical Modification (ICD-9-CM) coded discharge summaries and billing information. Patients’ demographic data were collected from the Israeli Central Bureau of Statistics and the Ministry of Internal Affairs, as previously reported [13]. The index date for inclusion in the study cohort was the date of the first PCI procedure performed for the indication of SCAD at RMC during the study period.

The inclusion criteria were: age>18 years at the index date, index PCI performed for an indication of SCAD, continued inclusion in the CHS at least 1 year prior and 1 year following the index PCI (unless the patient died within 1 year of the index date), adherence to DAPT for 6 months post-PCI (defined by documented prescriptions and procurement of both acetylsalicylic acid and clopidogrel [treatment with ticagrelor/prasugrel is permited only for ACS patients in Israel] following the index date) [14]. The exclusion criteria were: treatment with oral anticoagulation therapy at any time during the 6 months following the index date, pregnancy at the index date or during the following 6 months, a diagnosis of ACS or a PCI performed in the 6 months prior to the index date, a diagnosis of an ischemic or bleeding event (see appendix for detailed description) in the 6 months following the index date.

We hypothesised that since prolonging DAPT entails a trade-off between ischaemic and bleeding risks (reduction in ischemic risk with concomitant increase in bleeding risk), patients with a large absolute risk difference (ARD) between these opposing outcomes (ischaemia minus bleeding) are more likely to benefit from extending DAPT beyond 6 months. In contrast, patients with a low ARD are more likely to suffer harm from extending DAPT beyond 6 months. We further hypothesised that the ARD for each patient can be accurately predicted using a set of commonly available clinical characteristics. The three co-primary endpoints for this study were all-cause mortality; ischaemic events (primary efficacy endpoint): a composite endpoint defined as myocardial infarction (MI)/cerebrovascular accident (CVA)/PCI due to ACS, and major bleeding events that qualify at least as type 2 (e.g. requiring evaluation/intervention by a health care professional/admission) according to the bleeding academic research consortium (BARC) criteria (primary safety endpoint). Mortality was ascertained and cross referenced with the national mortality registry, Ischaemic and bleeding endpoints were first identified through the CHS data warehouse according to ICD-9 codes (see detailed description in the supplementary material) and in order to avoid abstraction/miscoding errors, ischaemic events were than verified against the outcome data in the RMC PCI registry (were outcomes are prospectively collected at 6 month’s intervals by trained research personnel through review of the patient’s online electronic medical record and/or telephone interview). Bleeding events (which are not routinely collected by the RMC PCI registry), and ischaemic events that were identified through ICD-9 codes but were not verified by the RMC PCI were independently adjudicated by the corresponding author (GW) through review of the patient’s electronic medical record.

The CHS’s institutional review board Review board ("Meir" Medical center, Kfar Saba, Israel) approved the study, the review board also waived the requirement for patients’ informed consent.

### Statistical methods

We developed a simplified risk score to approximate the expected ARD between ischaemic/bleeding events, and then examined its ability to identify subgroups of patients most likely to benefit/suffer from extending DAPT beyond 6 months post-PCI. This was done in five stages:

1. Separate prediction models were developed for the risk of ischaemic and major bleeding events for the 6–12 month period post-PCI: Baseline socioeconomic, demographic, clinical, and angiographic characteristics were compared between patients who did/did not experience ischaemic/major bleeding event during follow-up using the chi-square/Fisher’s exact test for categorical variables and the t-test/Mann-Whitney U test for continuous variables, as appropriate. Logistic regression was used to develop two prediction models–the first for ischaemic events and the second for major bleeding events. All baseline characteristics that showed a univariate association with ischaemic/bleeding events with a significance level of<0.3 were candidates for inclusion in the models. The final multivariable models were fitted using forward stepwise selection using the 0.05 significance level. Model discrimination was assessed using the c-statistic, and calibration was assessed using calibration plots and the Hosmer-Lemeshow goodness of fit for statistical significance.
2. The predicted ARD between ischaemic and major bleeding events (the difference between each participant’s predicted risk for ischaemic/major bleeding events according to the two prediction models mentioned above) was calculated.
3. A linear regression model was created to assess the association of each independent predictor of ischaemic/major bleeding risk with the predicted ARD between ischaemic/major bleeding events. This model used the predicted ARD as the outcome variable and all independent predictors of ischaemic/major bleeding events (identified by the separate ischaemic/major bleeding prediction models) as covariates.
4. A simplified risk score was developed to approximate the predicted ARD between ischaemic/major bleeding events by assigning an integer score to each independent predictor of ischaemic/major bleeding risk. The integer score for each variable was based on its ß coefficients in the linear regression model for predicting the ARD between ischaemic/major bleeding risk.
5. We examined the ability of stratification by predicted ARD/simplified risk score to identify patients likely to benefit versus suffer harm from extending the DAPT beyond 6 months. The cumulative incidence of outcomes (total mortality, composite ischaemic endpoint, composite bleeding endpoint) for patients treated with DAPT for 6 vs. 6+ months for the 6–12 month period post-PCI, were plotted by Kaplan-Meier curves and compared using the log-rank test, stratified by:
  - Predicted ARD between ischaemic/bleeding risk.
  - Simplified risk score for approximation of predicted ARD between ischaemic/bleeding risk.

The threshold for stratification was determined by examining the observed event rate in the multivariate model’s calibration plots. A two-tailed α of 0.05 was used to define the significance threshold for all comparisons. All statistical analyses were performed with SPSS software version 24.0 (IBM, Armonk, New-York, US) and R version 3.4.1 (R foundation for statistical computing, Vienna, Austria). The corresponding author (GW) had full access to all the data in the study and takes responsibility for its integrity and the data analysis.

## Results

**Fig 1:**
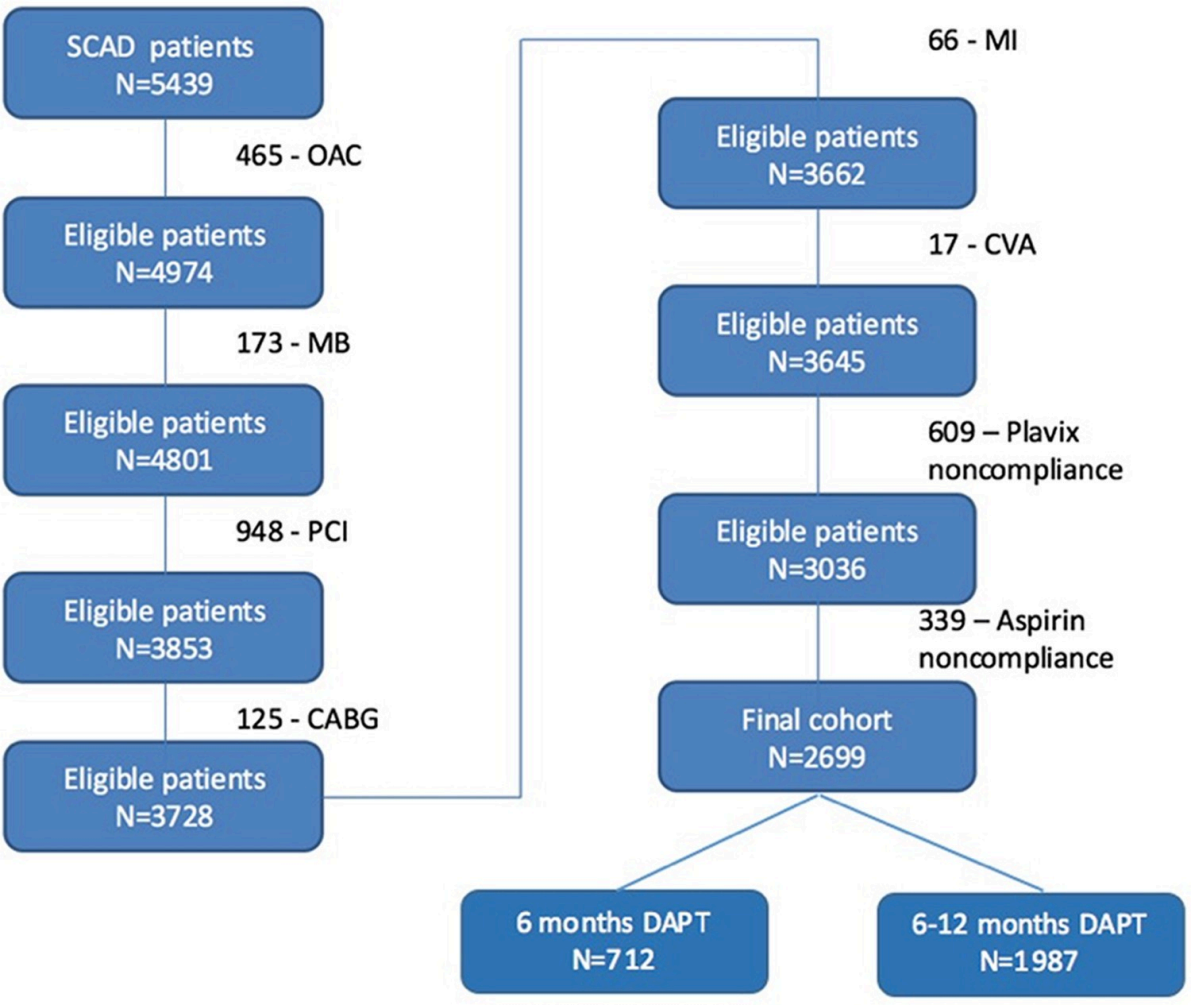
Cohort selection process. Reasons for exclusion of patients from study: CABG = coronary artery bypass graft surgery, CVA = cerebrovascular accident, MB = major bleeding, MI = myocardial infarction, OAC = oral anticoagulation, PCI = percutaneous coronary intervention. Other abbreviations: SCAD = stable coronary artery disease, DAPT = dual antiplatelet therapy.

From January 1^st^ 2004 to June 30^th^ 2016, 5439 patients underwent PCI due to SCAD at our medical centre; of these, 2699 were included in the final study cohort after applying all inclusion and exclusion criteria (Fig 1). During the 6 months follow up, 2.9% (78/2699) of patients experienced a primary ischaemic endpoint and 1.6% (43/2699) experienced a bleeding event. The baseline characteristics of patients who did vs. did not experience an ischaemic or bleeding event are shown in Table 1. Patients who experienced an ischaemic event had a higher prevalence of traditional cardiovascular risk factors (diabetes, hypertension, dyslipidaemia), prior evidence of established atherosclerotic disease (previous revascularization procedures, stroke), more complex coronary disease (left main and/or three vessel coronary disease), were less likely to be treated with drug eluting stents, and were less likely to receive statins. Patients who experienced a bleeding event were older, had worse renal function, and a higher prevalence of left main disease. Importantly, the DAPT duration did not show a statistically significant association with ischaemic or bleeding events. Although patients in the longer DAPT group had a numerically lower rate of ischemic events and higher rate of bleeding events, the difference was not statistically significant (Table 2).

**Table 1:** Patients’ characteristics according their clinical outcome status.

	Primary Ischaemic EP No (n = 2621)	Primary Ischaemic EP Yes (n = 78)	P value	Primary Bleeding EP No (n = 2656)	Primary Bleeding EP Yes (n = 43)	P value
**Demographics**						
Age (years)	65.52±10.87	66.56±10.63	0.33	65.48±10.83	70.90±11.46	<0.01
Male gender	2016(77.8%)	89(82.4%%)	0.26	2068(77.9%)	37(86%)	0.20
BMI (kg/m^2^)	28.56±4.34	28.81±5.16	0.10	28.56±4.34	28.81±5.16	0.10
EF (%)	56.13±7.79	53.27±10.18	0.06	56.±7.96	55.53±8.96	0.80
**Comorbidities**						
Diabetes at PCI	1148(44.3%)	60(55.6%)	0.02	1188(44.7%)	20(46.5%)	0.82
Hypertension at PCI	2098(81%)	98(90.7%)	0.01	2160(81.3%)	36(83.7%)	0.69
Dyslipidaemia at PCI	2408(92.9%)	106(98.1%)	0.04	2472(93.1%)	42(97.7%)	0.24
Smoking status at PCI			0.92			0.67
Non-smoker	1094(51.5%)	41(50.6%)		1079(5./4%)	18(46.2%)	
Former	636((29.9%)	23(28.4%)		561(27.2%)	13(33.3%)	
Active	394(18.5%)	17(21%)		419(20.3%)	8(20.5%)	
S/P AMI	473(18.3%)	24(22.2%)	0.30	487(18.3%)	10(23.3%)	0.41
S/P PCI	1154(44.5%)	63(58.3%)	<0.01	1194(45%)	23(53.5%)	0.27
S/P CABG	276(10.7%)	18(16.7%)	0.05	289(10.9%)	5(11.6%)	0.88
S/P CVA	362(14%)	35(32.4%)	<0.01	390(14.7%)	7(16.3%)	0.77
Atrial fibrillation	210(8.1%)	14(13%)	0.07	217(8.2%)	7(16.3%)	0.06
Malignancy^*^	386(14.9%)	14(13%)	0.58	393(14.8%)	7(16.3%)	0.79
**Medications**						
Oral hypoglycaemic	828(32%)	34(31.5%)	0.92	852(32.1%)	10(23.3%)	0.22
Insulin	284(11%)	22(20.4%)	<0.01	301(11.3%)	5(11.6%)	0.95
NSAIDS	17(0.7%)	0(0%)	0.40	17(0.6%)	0(0%)	0.60
PPI	863(33.3%)	42(38.9%)	0.22	891(33.5%)	14(32.6%)	0.89
H2RA	276(10.7%)	15(13.9%)	0.29	286(10.8%)	5(11.6%)	0.86
Anti-hypertensive treatment	2398(92.2%)	99(91.7%)	0.89	2447(92.1%)	41(95.3%)	0.44
Statins	2374(91.6%)	88(81.5%)	<0.01	2421(91.2%)	41(95.3%)	0.34
**Laboratory results**						
Haemoglobin at PCI (mg/dL)	13.70± 1.53	13.58±1.49	0.07	13.70± 1.53	13.38±1.78	0.29
Platelets at PCI (x10^9^)	235±68	221±57	0.12	235±67	224±71	0.40
INR at PCI	1.00±0.14	1.01±0.92	0.32	1.00±0.12	1.17±0.88	0.27
Albumin at PCI (g/dL)	4.312±0.31	4.314±0.33	0.98	4.312±0.31	4.227±0.24	0.37
GFR at PCI (mi/min/1.73m^2^)	63.17.±22.54	61.23±30.77	0.11	66.11.±22.96	57.15±19.85	0.01
LDL-C at PCI(mg/d)	96.73.±33.29	99.01.±37.11	0.56	95.38±31.2	94.17.20±34.9	0.87
HDL-C at PCI(mg/d)	42.57±11.0	41.21±9.5	0.28	43.44±11.10	44.30±11.9	0.74
HbA1C (%)	7.37±1.99	7.16±2.14	0.65	7.36±1.78	6.36±0.58	0.33
**Angiographic data**						
Radial	1138(43.4%)	29(37.6%)	0.16	1152(43.3%)	15(34.9%)	0.07
Number Vessel Disease			0.01			0.76
1	614(23.7%)	12(11.5%)		618(23.3%)	8(18.6%)	
2	877(33.8%)	39(36.1%)		899(33.8%)	17(39.5%)	
3	1077(41.6%)	57(52.8%)		1116(42%)	18(41.9%)	
LM disease	46(1.8%)	5(4.6%)	0.03	48(1.8%)	3(7%)	0.01
Proximal LAD disease	463(17.9%)	12(11.1%)	0.07	466(17.5%)	9(20.9%)	0.56
Any proximal main vessel disease	1145(44.3%)	47(43.9%)	0.94	1171(44.2%)	21(48.8%)	0.55
Chronic Total Occlusion	92(3.6%)	0(0%)	0.12	90(3.4%)	2(4.7%)	0.61
Bifurcation	114(4.4%)	2(1.9%)	0.39	114(4.3%)	2(4.7%)	0.63
Calcifications	147(5.7%)	10(9.3%)	0.29	151(5.7%)	6(14%)	0.07
Overall stent length (mm)	30.06±19.36	30.16±17.7	0.93	30.06±19.16	26.86±16.2	0.28
Maximal stent length (mm)	19.90±6.8	20.03±7.5	0.86	19.93±7.0	18.76±6.3	0.25
Mean stent size (mm)	2.96±0.45	3.01±0.51	0.24	2.96±0.46	3.10±0.49	0.05
Min stent size (mm)	2.84±0.48	2.90±0.56	0.36	2.85±0.49	2.97±0.57	0.1
Max stent size (mm)	3.08±0.49	3.14±0.54	0.21	3.08±0.49	3.23±0.50	0.04
Stent type			0.42			0.53
BMS	964(36.8%)	37(47.4%)		989(37.2%)	12(27.9%)	
PES	84(3.2%)	2(1.4%)		85(3.2%)	1(2.3%)	
SES	449(17.1%)	15(19.2%		457(17.2%)	7(16.3%)	
EES	462(17.6%)	9(11.5%)		463(17.4%)	8(18.6%)	
ZES	373(14.2%)	10(12.8%)		373(14.0%)	10(23.3%)	
BES	158(6.0%)	2(2.6%)		156(5.9%)	4(9.3%)	
Mixed DES	131(5.0%)	3(3.8%)		133(5.0%)	1(2.3%)	
**DAPT duration**						
6+months DAPT	1936(73.7%)	51(64.1%)	0.27	1951(73.3%)	36(83.7%)	0.15

AMI = acute myocardial infarction, BES = Biolimus eluting stent, BMI = body mass index, CABG = coronary artery bypass graft surgery, BMS = bare metal stent, CVA = cerebrovascular accident, DAPT = dual antiplatelet therapy, DES = drug eluting stent, EES = Everolimus eluting stent, EF = ejection fraction, EP = endpoint, GFR = glomerular filtration rate, HbA1C = haemoglobin A1C, HDL-C = high density lipoprotein cholesterol, H2RA = histamine 2 receptor antagonists, INR = international normalized ratio, LAD = left anterior descending, LDL-C = low density lipoprotein cholesterol, LM = left main, NSAIDS = non-steroidal anti-inflammatory drugs, PCI = percutaneous coronary intervention, PES = Paclitaxel eluting stent, PPI = proton pump inhibitors, SES = Sirolimus eluting stent, ZES = Zatarolimus eluting stent.

*Diagnosed within 1 year prior to PCI.

**Table 2:** Summary of endpoints by DAPT duration.

Outcome	Overall (n = 2699)	Short DAPT (n = 712)	Long DAPT (n = 1987)	P value
Primary ischaemic EP^*^	78(2.9%)	27(3.8%)	51(2.6%)	0.117
Death	35(1.3%)	13(1.8%)	22(1.1%)	0.175
AMI	31(1.1%)	12(1.7%)	19(0.9%)	0.149
CVA	16(0.6%)	4(0.6%)	12(0.6%)	1
PCI for ACS	34(1.3%)	11(1.5%)	23(1.2%)	0.435
Major Bleeding	43(1.6%)	7(1.0%)	36)1.8%)	0.162

* AMI/CVA/PCI for ACS. ACS = acute coronary syndromes, AMI = acute myocardial infarction, CVA = cerebrovascular accident, EP = endpoint, PCI = percutaneous coronary intervention

Using a multivariable logistic regression, four variables (any history of symptoms related to congestive heart failure/ documented ejection fraction≤30%, left main or three vessel coronary disease, status post [s/p] PCI, and s/p CVA) were found to be independent predictors of ischaemic events and two variables (age>75 years and baseline haemoglobin<10 mg/dL) were found to be independent predictors of major bleeding events (Table 3).

**Table 3:** Independent predictors of ischaemic and bleeding events after multivariate adjustment.

Ischaemia model				Major Bleeding model			
Variable	OR	95% CI	PV	Variable	0R	95% CI	PV
CHF/EF<30%	2.27	1.34–3.83	0.002	Age>75	3.10	1.64–5.86	<0.001
LM/3VD	1.48	1.10–2.36	0.041	Hb<10	6.41	2.35–17.46	<0.001
S/P PCI	1.91	1.17–3.12	0.009				
S/P CVA	2.48	1.49–4.13	<0.001				

CHF = congestive heart failure, CVA = cerebrovascular accident, EF = ejection fraction, Hb = haemoglobin, LM = left main, PCI = percutaneous coronary intervention, S/P = status post, 3VD = triple vessel disease

**Fig 2:**
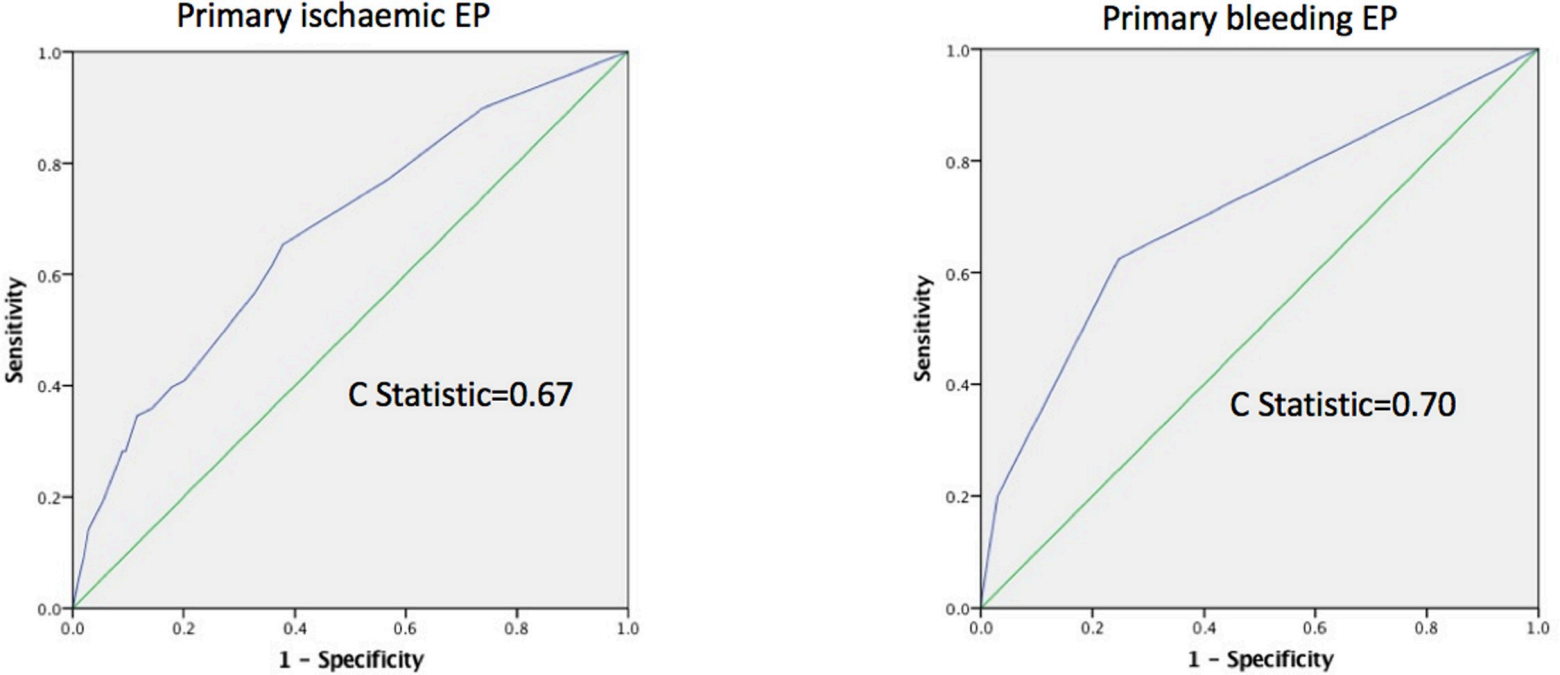
ROC curves for prediction models. ROC curves and c-statistics of the ischaemic (left) and bleeding (right) prediction models.

**Fig 3:**
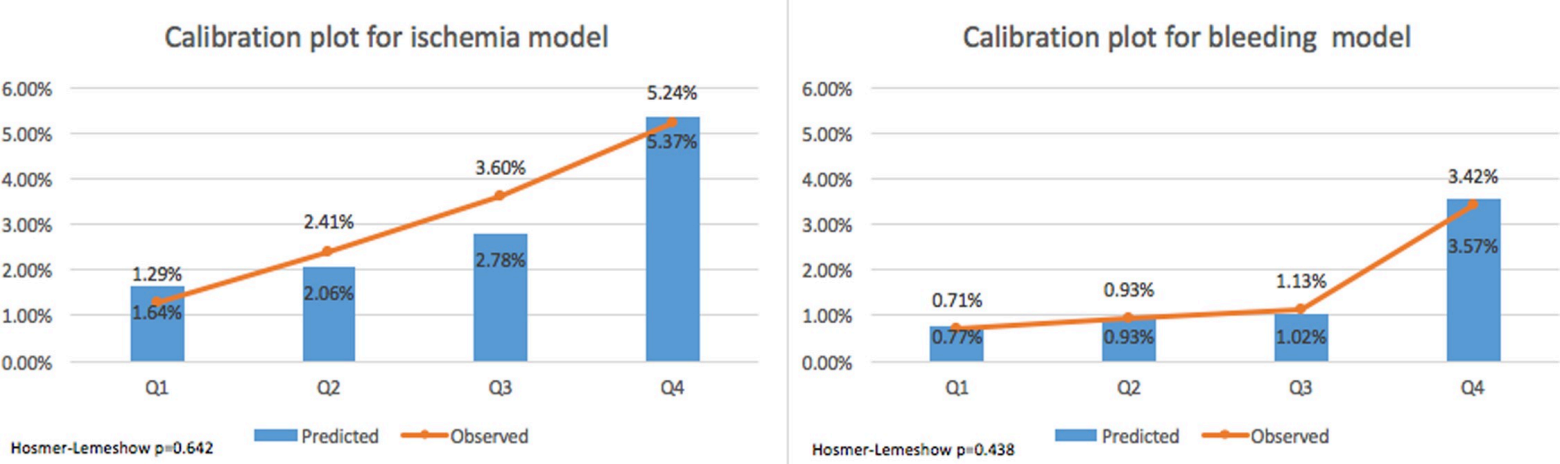
Calibration plots for the prediction models. Calibration plots of the ischaemia (left) and bleeding (right) prediction models, showing the predicted (blue bars) and observed (orange line and dots) rate of events stratified by the quartiles of the predicted risk.

The ROC curves for the ischaemia and major bleeding prediction models are shown in Fig 2, the c-statistics were 0.67 and 0.70, respectively. Calibration plots for each prediction model, stratified by quartiles of predicted risk are shown in Fig 3. The calibrations of the ischaemic and major bleeding risk models were good (p = 0.642 and p = 0.438 for the Hosmer-Lemeshow goodness of fit of the ischaemia and major bleeding models, respectively). For both endpoints, the most significant increase in risk was between the third and fourth quartile. Therefore, we used this threshold for stratification of the cohort into high/low predicted ARD (ischaemic minus major bleeding) for the purpose of examining the ability of our risk prediction model and risk score to identify patients likely to benefit/suffer from extending the DAPT duration (see below).

**Fig 4:**
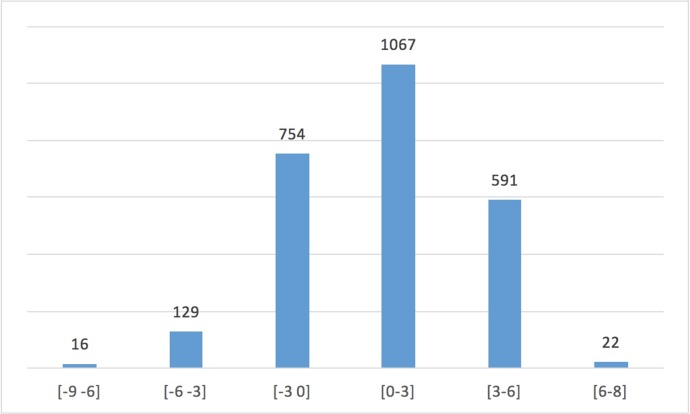
Distribution of risk score values in the study cohort. Distribution of risk score values in the study cohort.

The results of the linear regression model fitted to approximate the predicted ARD of ischaemic/major bleeding events showed good correlation with the actual predicted ARD values (Pearson’s r = 0.915, R^2^ = 0.837). Each covariate in the model (the independent predictors of ischaemic/major bleeding events identified by the separate logistic regression models) was highly statistically significant (p<0.001 for all six covariates). The integer scores for each variable included in the final clinical risk score are shown in Table 4. The overall scores ranged from -9 to +8, and the distribution of the score is shown in Fig 4. The value of the 75^th^ percentile was 3.

**Table 4:** Translation of the risk model into an integer score.

Variable	score
CHF/EF<30%	+2
LM/3VD	+1.5
S/P PCI	+2
S/P CVA	+2.5
Age>75 years	-3
Hb<10	-6
Total score range	-9 to 8

CHF = congestive heart failure, CVA = cerebrovascular accident, EF = ejection fraction, Hb = haemoglobin, LM = left main, PCI = percutaneous coronary intervention, S/P = status post, 3VD = triple vessel disease

**Fig 5:**
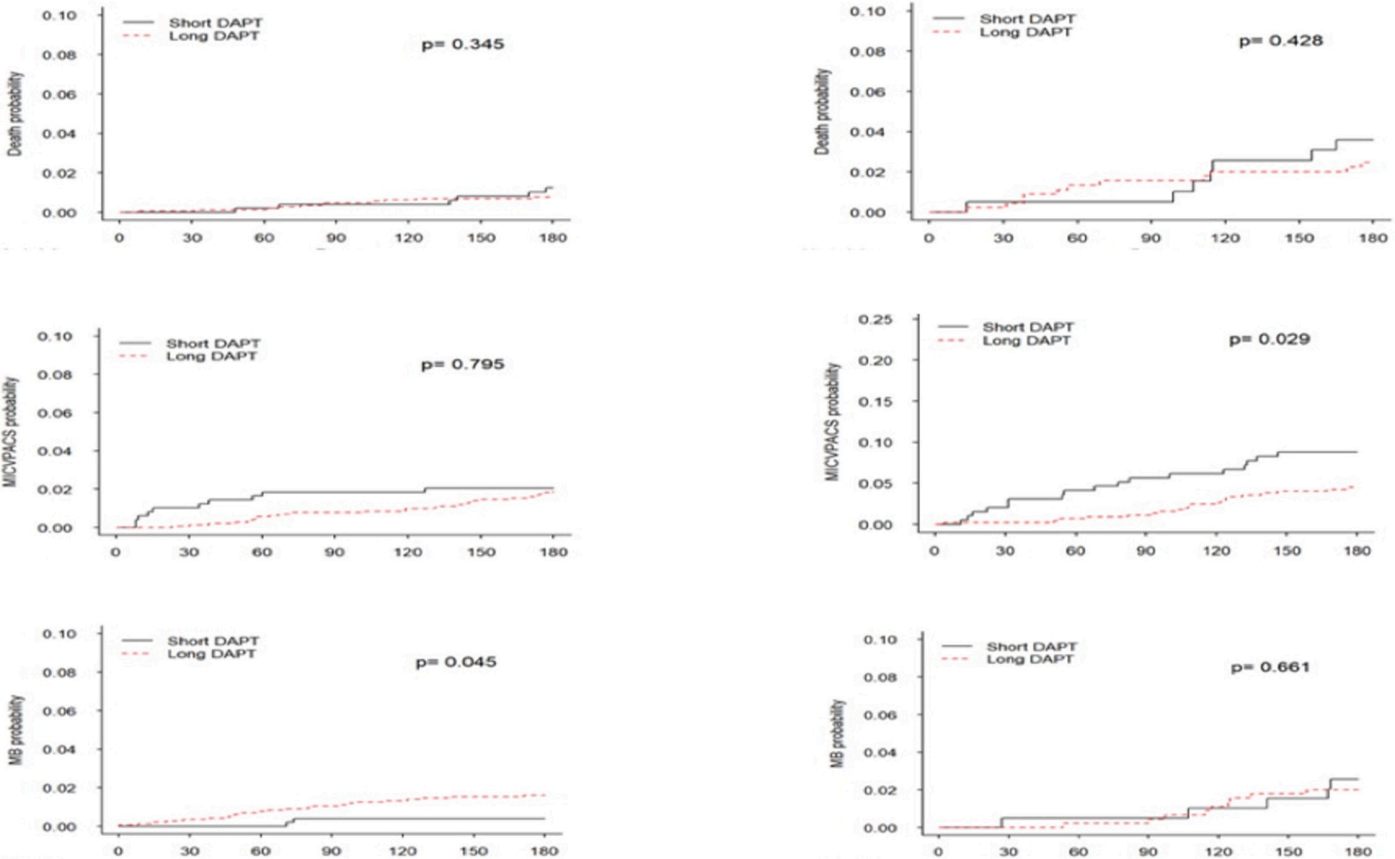
Kaplan-Meier curves for clinical outcomes according to predicted ARD stratum. Kaplan Meier curves for the overall mortality (top row), composite ischaemic endpoint (middle row), and bleeding (bottom row) of patients treated with shorter (6 months) vs. longer (6–12 months) DAPT, stratified by quartiles of the predicted ARD between ischemic and bleeding events–lower three quartiles (left column) and upper quartile (right column). DAPT = dual antiplatelet therapy, MB = major bleeding.

Kaplan Meier curves comparing a longer vs. shorter DAPT, stratified by the predicted ARD between ischaemia and major bleeding events (Q 1–3 and Q4), are shown in Fig 5. In both strata, there was no difference in overall mortality between patients treated with longer vs. shorter DAPT. However, for ischaemic and major bleeding events, DAPT duration had a differential effect in the two strata. In the bottom three quartiles, a longer DAPT duration was associated with an increased risk of major bleeding, but a similar risk for ischaemia; whereas, in the upper quartile, a longer DAPT duration resulted in a lower ischemic risk and no increase in major bleeding events. For the ARD, in the lower three quartiles, a longer DAPT duration resulted in an absolute increase of 1% (95% CI: -1.4 to +3.4%) in the risk for overall clinical (ischaemic + major bleeding) events (number needed to treat [for harm] 100). In the upper quartile, a longer DAPT duration resulted in an absolute reduction of 4.8% (95% CI -11.6 to +2.0%) in the overall risk (number needed to treat 20) (Table 5).

**Table 5:** Outcomes of patients treated with short vs. long DAPT stratified by the absolute predicted risk difference between ischaemic and bleeding events.

	Risk for ischaemia+bleeding	95%CI	ARD with long DAPT
Q1-3 short DAPT	2.5%	1.1%-3.9%	1(-1.4 to +3.4)%
Q1-3 long DAPT	3.5%	2.5%-4.5%	
Q4 short DAPT	11.3%	6.8%-15.8%	-4.8(-11.6 to +2.0)%
Q4 long DAPT	6.5%	4.2%-8.8%	

ARD = absolute risk difference, CI = confidence interval, DAPT = dual antiplatelet therapy, MB = major bleeding

**Fig 6:**
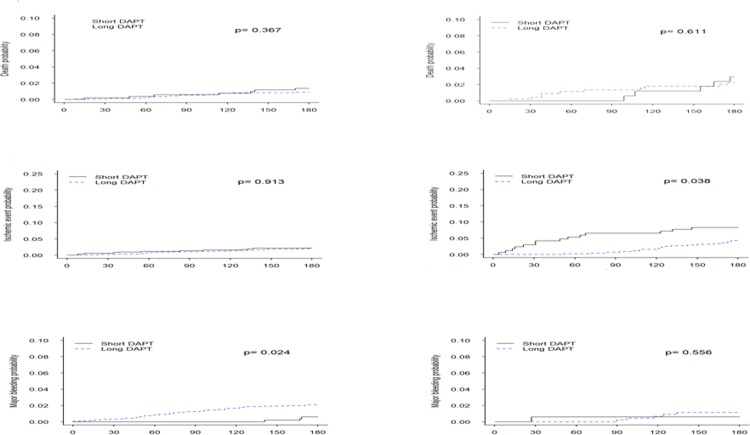
Kaplan-Meier curves for clinical outcomes according to simplified risk score stratum. Kaplan Meier curves for the overall mortality (top row), composite ischaemic endpoint (middle row), and bleeding (bottom row) of patients treated with shorter (6 months) vs. longer (6–12 months) DAPT, stratified by quartiles of the risk score–lower three quartiles (left column) and upper quartile (right column). DAPT = dual antiplatelet therapy.

When the cohort was stratified according to the simplified clinical risk score (with a threshold of three separating the bottom three quartiles from the upper quartile), the results were consistent. There was no difference in overall mortality according to DAPT duration, but there was an advantage for longer DAPT duration in terms of ischaemia with no concomitant increased risk for major bleeding in the upper quartile and an advantage for shorter DAPT was found in terms of reduction of major bleeding with no change in ischaemic risk in the bottom three quartiles (Fig 6).

**Fig 7:**
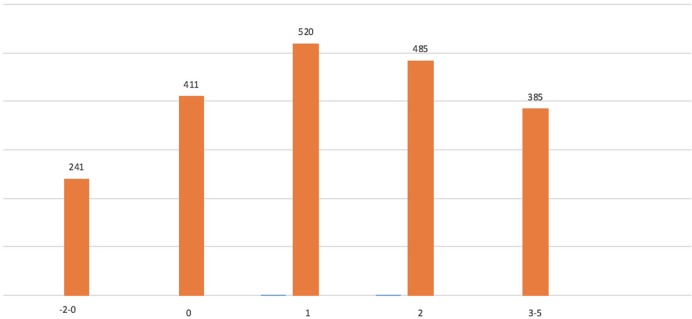
Distribution of DAPT scores in the study cohort. Distribution of DAPT scores in the study cohort.

We were then able to calculate the DAPT score in 76% (2042/2699) of the patients included in our cohort, with a limitation of not having data on PCIs performed within saphenous vein grafts. The median score was 1, the distribution of DAPT score in our cohort is shown in Fig 7. The c-statistics of the DAPT score in our cohort were 0.60 for ischemic events and 0.50 for bleeding events.

## Discussion

Our results showed that for patients who underwent PCI due to SCAD and completed 6 months of DAPT without having experienced any ischaemic/bleeding events, a structured decision tool based on a few clinical characteristics can identify those most likely to benefit from extending the duration of DAPT past 6 months, similar to what was previously shown for a PCI population completing 1 year of DAPT [5]. It is now well-acknowledged that the duration of DAPT should be individualised and tailored to each patient, as bleeding events can be associated with a longer DAPT duration. The optimal DAPT duration may range from 3–6 months to 30 months post-PCI, depending on the characteristics of the patient [15]. Current guidelines allow cardiologists very broad leeway in determining the DAPT duration, and recommend basing this decision on the physician’s assessment of the patient’s ischaemic and bleeding risks. The main problem is that this assessment is obviously very subjective, and until recently, there were no validated tools to quantify these risks and aid in making this clinical decision. The DAPT and PRECISE-DAPT scores are currently the only such tools, and are both recommended by the latest European guidelines [3]. The DAPT score was developed to identify patients who are likely to benefit from extending DAPT beyond 1 year, and as such, contains components evaluating both ischemic and bleeding risk factors. It has two main limitations: 1) it is only relevant to patients who have completed 1 year of DAPT; therefore, it not suitable to making the decision of whether to continue DAPT beyond 6 months, which is when this should be addressed in the SCAD population and 2) the external validity is unclear. As part of its development process, the score was externally validated using the population of the PROTECT trial, and despite c-statistics for the ischaemic and bleeding models that were similar to those of the derivation cohort, it was not able to identify those at higher bleeding risk (while maintaining the ability to identify those at higher ischaemic risk) [5]. Although the DAPT score’s ability to predict future ischaemic events, irrespective of DAPT treatment duration has been externally validated in a large cohort [16], It’s limited external validity to identify patients likely to benefit from longer/shorter DAPT duration has been consistently shown using cohorts of patients from randomized trials [8,11,17], registry populations [17], or a mixture of both [18]. The same limitation applies to the PRECISE-DAPT score, as its ability to identify patients likely to experience clinical benefit/harm from extended DAPT duration has not undergone external validation [6].

Our risk score addresses one of the limitations of the DAPT score, as SCAD patients can be assessed for risk at 6 months post-PCI. Potentially (after appropriate validation), the two scores can be used consecutively, in a complementary fashion, for SCAD patients undergoing PCI–to choose suitable patients for continuation of DAPT beyond 6 months post-PCI, and then using the DAPT score at 12 months post-PCI to identify those likely to benefit from extending DAPT even further. This sort of a staged decision making process will be more in line with current guidelines regarding DAPT duration in the SCAD population.

Another novel aspect of our score, is that it is based on a population from a clinical registry, rather than an RCT. It is well-recognised that the populations of RCTs tend to differ from the average population seen in daily practice [19]. Consequently, the Achilles’ heel of risk scores based exclusively on an RCT population is the limited external validity. Developing such scores from registries of all comers patients has its own limitations (see below), but can potentially improve the external validity. This stems from two main reasons: higher similarity to the target population, and allowing the development of risk scores based on cohorts that are homogenous in terms of clinical presentation (i.e. SCAD/ACS/MI), given the well-recognised association of clinical presentation with benefit from longer DAPT duration [3]. Unfortunately, all-comers registries usually contain data on medical treatment at baseline only and prospective data only on clinical endpoints. Hence, the most available source for data on patients treated with DAPT for different, but known, durations are RCTs comparing different DAPT durations. To circumvent this, we cross-referenced and combined two separate registries–a dedicated PCI registry and an administrative HMO-type database that contains pharmacological data during follow up. The developed risk score has many limitations (as acknowledged below), most notably, we did not validate our risk score in a separate cohort so we believe our results should be viewed as a proof-of-concept for the potential benefits of using all-inclusive PCI registries to create risk scores for determining the DAPT duration, rather than a testament to the clinical value of the risk score we developed.

Our study has several other limitations. Since the DAPT duration in our cohort was not randomly assigned, we cannot exclude residual confounding that may have influenced our results. In addition, we included patients undergoing PCI at a single centre and over a long period, thus our results have limited generalizability and require validation from more diverse clinical setting, although as seen from the data presented in Table 1 –the characteristics of our patients are similar to those of SCAD patients undergoing PCI reported elsewhere [20]. this also resulted in several procedural characteristics (most notably the use of bare metal stents and the radial approach) differed from current standards; however, the ischaemic endpoints we chose are not affected by stent type [21] and similarly, bleeding events beyond 6 months post-PCI should not be influenced by the access route. We did not collect data on previous bleeding events (prior to the index PCI), which has been found to be associated with bleeding risk under DAPT and is included in the PRECISE-DAPT score [6], Due to the administrative requirementsof the Israeli healthcare system, our cohort included only patients treated with clopidogrel (which is still the P_2_Y_12_ inhibitor of choice in SCAD). Because the follow up period was only 6 months and the study included SCAD patients only, our prediction models are based on a small number of outcomes (that are representative of this population when compared to clinical trials reporting outcomes in the year following PCI in populations with a high representation of SCAD patients) [22–24] and the discrimination ability of our model was modest for both ischaemic and bleeding events (though similar to the discrimination abilities of currently recommended risk scores). Finally, our endpoints were primarily identified through ICD-9 CM codes, which are prone to abstraction and miscoding errors, but mortality was ascertained beyond doubt (against a national registry), ischaemic events were verified against a registry that performs a prospective adjudication of outcomes, and bleeding events were adjudicated independently by the investigators, so the main concern regarding outcomes is under reporting rather than lack of validity. As mentioned above–the rate of both ischaemic and bleeding events was similar to that reported in prospective randomised trials, which supports the validity of our outcomes. Another issue worth mentioning is that we used the ARD between ischaemic and bleeding endpoint for the purpose of stratifying our cohort into risk groups, which implies that ischaemic and bleeding events have the same clinical impact, a concept used by both Yeh et al [5], as well as Costa et al [6], but has not been extensively studied. There is, however data showing that at least in terms of their effect on subsequent overall mortality–ischaemic and bleeding events have similar impact [25].

## Conclusions

In a population of SCAD patients who completed 6 months of DAPT post-PCI with no bleeding/ischaemic events, a risk score for ischaemic and bleeding events identified patients likely to gain from continuing/stopping DAPT with good calibration and fair discrimination. This decision tool requires external validation, and we hope our results will spur further efforts to develop better tools to assist with making decisions on DAPT duration.

## Supporting information

S1 Fig Definitions of ICD-9-CM codes used for identifying clinical endpoints. (DOCX)

## Abbreviations

ARD: absolute risk difference
ACS: acute coronary syndromes
CVA: cerebrovascular accident
DAPT: dual antiplatelet therapy
MI: myocardial infarction
PCI: percutaneous coronary interventions
SCAD: stable coronary artery disease

## References

[1] Bittl JA, Baber U, Bradley SM and Wijeysundera DN. Duration of Dual Antiplatelet Therapy: A Systematic Review for the 2016 ACC/AHA Guideline Focused Update on Duration of Dual Antiplatelet Therapy in Patients With Coronary Artery Disease: A Report of the American College of Cardiology/American Heart Association Task Force on Clinical Practice Guidelines. J Am Coll Cardiol. 2016;68:1116–39. 10.1016/j.jacc.2016.03.512 27036919

[2] Levine GN, Bates ER, Bittl JA, Brindis RG, Fihn SD, Fleisher LA, et al 2016 ACC/AHA Guideline Focused Update on Duration of Dual Antiplatelet Therapy in Patients With Coronary Artery Disease: A Report of the American College of Cardiology/American Heart Association Task Force on Clinical Practice Guidelines. J Am Coll Cardiol. 2016;68(10):1082–115. 10.1016/j.jacc.2016.03.513 27036918

[3] Valgimigli M, Bueno H, Byrne RA, Collet JP, Costa F, Jeppsson A, et al 2017 ESC focused update on dual antiplatelet therapy in coronary artery disease developed in collaboration with EACTS: The Task Force for dual antiplatelet therapy in coronary artery disease of the European Society of Cardiology (ESC) and of the European Association for Cardio-Thoracic Surgery (EACTS). Eur Heart J. 2017. 10.1093/ejcts/ezx334 29045581

[4] Valgimigli M, Costa F, Byrne R, Haude M, Baumbach A, Windecker S. Dual antiplatelet therapy duration after coronary stenting in clinical practice: results of an EAPCI survey. EuroIntervention. 2015;11:68–74. 10.4244/EIJV11I1A11 25982650

[5] Yeh RW, Secemsky EA, Kereiakes DJ, Normand SL, Gershlick AH, Cohen DJ, et al Development and Validation of a Prediction Rule for Benefit and Harm of Dual Antiplatelet Therapy Beyond 1 Year After Percutaneous Coronary Intervention. JAMA. 2016;315(16):1735–49. 10.1001/jama.2016.3775 27022822 PMC5408574

[6] Costa F, van Klaveren D, James S, Heg D, Raber L, Feres F, et al Derivation and validation of the predicting bleeding complications in patients undergoing stent implantation and subsequent dual antiplatelet therapy (PRECISE-DAPT) score: a pooled analysis of individual-patient datasets from clinical trials. Lancet. 2017;389(10073):1025–34. 10.1016/S0140-6736(17)30397-5 28290994

[7] Lenzen MJ, Boersma E, Bertrand ME, Maier W, Moris C, Piscione F et al Managemebt and outcome of patients with established coronary artery disease: the Euru HeartSurvey on coronary revascularization. Eur Heart J. 2005; (12):1169–79. 10.1093/eurheartj/ehi238 15802360

[8] Harada Y, Michel J, Lohaus R, Mayer K, Emmer R, Lahmann AL, et al Validation of the DAPT score in patients randomized to 6 or 12 months clopidogrel after predominantly second-generation drug-eluting stents. Thromb Haemost. 2017;117(10). 10.1160/TH17-02-0101 28783201

[9] Schulz-Schupke S, Byrne RA, Ten Berg JM, Neumann FJ, Han Y, Adriaenssens T, et al ISAR-SAFE: a randomized, double-blind, placebo-controlled trial of 6 vs. 12 months of clopidogrel therapy after drug-eluting stenting. Eur Heart J. 2015;36(20):1252–63. 10.1093/eurheartj/ehu523 25616646

[10] Valgimigli M, Campo G, Monti M, Vranckx P, Percoco G, Tumscitz C, et al Short- versus long-term duration of dual-antiplatelet therapy after coronary stenting: a randomized multicenter trial. Circulation. 2012;125(16):2015–26 10.1161/CIRCULATIONAHA.111.071589 22438530

[11] Piccolo R, Gargiulo G, Franzone A, Santucci A, Ariotti S, Baldo A, et al Use of the Dual-Antiplatelet Therapy Score to Guide Treatment Duration After Percutaneous Coronary Intervention. Ann Intern Med. 2017;167(1):17–25. 10.7326/M16-2389 28605779

[12] Bental T, Assali A, Vaknin-Assa H, Lev EI, Brosh D, Fuchs S, et al A comparative analysis of major clinical outcomes using drug-eluting stents versus bare-metal stents in a large consecutive patient cohort. Catheter Cardiovasc Interv. 2010;76(3):374–80. 10.1002/ccd.22507 20839351

[13] Leibowitz M, Karpati T, Cohen-Stavi CJ, Feldman BS, Hoshen M, Bitterman H, et al Association Between Achieved Low-Density Lipoprotein Levels and Major Adverse Cardiac Events in Patients With Stable Ischemic Heart Disease Taking Statin Treatment. JAMA Intern Med. 2016;176(8):1105–13. 10.1001/jamainternmed.2016.2751 27322095

[14] Singer SR, Hoshen M, Shadmi E, Leibowitz M, Flaks-Manov N, Bitterman H, et al EMR-based medication adherence metric markedly enhances identification of nonadherent patients. Am J Manag Care. 2012;18(10):e372–7. 23145845

[15] Binder RK and Luscher TF. Duration of dual antiplatelet therapy after coronary artery stenting: where is the sweet spot between ischaemia and bleeding? Eur Heart J. 2015;36:1207–11. 10.1093/eurheartj/ehv103 25838437

[16] Yoshikawa Y, Shiomi H, Watanabe H, Natsuki H, Kondo T, Tamura et al Validating Utility of DAPT Score in a Large Pooled Cohort from Three Japanese PCI Studies.Circulation. 2018;137:551–62 10.1161/CIRCULATIONAHA.117.028924 28982692

[17] Wong YTA, Kang DY, Ahn JM, Do U, Kwon O, Lee K et al External validation of DAPT score in two large cohorts. J Am Coll Cardiol. 2017; 69 (11 Supplement)34357–7 (abstract).

[18] Yoshikawa Y, Shiomi H, Watanabe M, Natsuki H, Kondo T, Tamura T et al Application of DAPT score to predict ischaemic and bleeding events in patients who underwent drug-eluting stent implantation: a landmark analysis of large pooled cohort. EurHeart J. 2017;38 (1- supplement 1) 611–2(abstract).

[19] Steg PG, Lopez-Sendon J, Lopez de Sa E, Goodman SG, Gore JM, Anderson FA Jr., et al External validity of clinical trials in acute myocardial infarction. Arch Intern Med. 2007;167(1):68–73. 10.1001/archinte.167.1.68 17210880

[20] Hannan EL, Samadashvili Z, Cozzens K, Walford G, Jacobs AK, Holmes DR Jr et al Comparative outcomes for patients who do and do not undergo percutaneous coronary intervention for stable coronary artery disease in New York. Circulation. 2012; 125:1870–9. 10.1161/CIRCULATIONAHA.111.071811 22441935

[21] Bangalore S, Kumar S, Fusaro M, Amoroso N, Attubato MJ, Feit F, et al Short- and long-term outcomes with drug-eluting and bare-metal coronary stents: a mixed-treatment comparison analysis of 117 762 patient-years of follow-up from randomized trials. Circulation. 2012;125(23):2873–91. 10.1161/CIRCULATIONAHA.112.097014 22586281

[22] Gwon HC, Hahn JY, Park KW, Song YB, Chae IH, Lim DS et al Six-month versus 12-month dual antiplatelet therapy after implantation of drug-eluting stents: the Efficacy of Xience/Promus Versus Cypher to Reduce Late Loss After Stenting (EXCELLENT) randomized, multicenter study. Circulation. 2012;125(3):505–13. 10.1161/CIRCULATIONAHA.111.059022 22179532

[23] Kim BK, Hong MK, Shin DH, Nam CM, Kim JS, Ko YG et al A new strategy for discontinuation of dual antiplatelet therapy: the RESET Trial (REal Safety and Efficacy of 3-month dual antiplatelet Therapy following Endeavor zotarolimus-eluting stent implantation). J Am Coll Cardiol. 2012; 60(15):1340–8. 10.1016/j.jacc.2012.06.043 22999717

[24] Feres F, Costa RA, Abizaid A, Leon MB, Marin-Neto JA, Botelho RV et al Three vs twelve months of dual antiplatelet therapy after zotarolimus-eluting stents: the OPTIMIZE randomized trial. JAMA. 2013;310(23):2510–22. 10.1001/jama.2013.282183 24177257

[25] Baber U, Dangas G, Chandrasekhar J, Sartori S, Steg PG, Cohen DJ et al timre Dependent Associations Between Actionable Bleeding, Coronary Thrombotic Events, and Mortality Following Percutaneous Coronary Intervention: Results From the PARIS Registry. JACC Cardiovasc Interv. 2016;9:1349–57. 10.1016/j.jcin.2016.04.009 27388822

