# Supplementary material for: Development of a risk score for predicting the benefit versus harm of extending dual antiplatelet therapy beyond 6 months following percutaneous coronary intervention for stable coronary artery disease
Source: PLoS One. 2019 Feb 14;14(2):e0209661. doi: 10.1371/journal.pone.0209661 (PMC6375573; doi:10.1371/journal.pone.0209661)
Supplement: S1 Fig — (DOCX) [file pone.0209661.s001.docx]

Supplementary material

Definitions of ICD-9-CM codes used for identifying clinical endpoints

Bleeding:

- Intracranial hemorrhage – 430 (SUBARACHNOID HEMORRHAGE), 431 (INTRACEREBRAL HEMORRHAGE), 432.0(NONTRAUMATIC EXTRADURAL HEMORRHAGE), 432.1(SUBDURAL HEMORRHAGE), 432.9(UNSPECIFIED INTRACRANIAL HEMORRHAGE);
- Intraocular hemorrhage- 362.81(RETINAL HEMORRHAG), 363.61(CHOROIDAL HEMORRHAGE UNSPECIFIED), 363.62(EXPULSIVE CHOROIDAL HEMORRHAGE), 379.23(VITREOUS HEMORRHAGE), 360.43(HEMOPHTHALMOS EXCEPT CURRENT INJURY);
- Retroperitoneal bleeding - 459.0;
- Gastrointestinal bleeding - 578.9(HEMORRHAGE OF GASTROINTESTINAL TRACT UNSPECIFIED),530.82(ESOPHAGEAL HEMORRHAGE), 531.00 ,531.01(ACUTE GASTRIC ULCER WITH HEMORRHAGE WITH OBSTRUCTION), 531.20(ACUTE GASTRIC ULCER WITH HEMORRHAGE AND PERFORATION WITHOUT OBSTRUCTION),531.21(ACUTE GASTRIC ULCER WITH HEMORRHAGE AND PERFORATION WITH OBSTRUCTION) ,531.40(CHRONIC OR UNSPECIFIED GASTRIC ULCER WITH HEMORRHAGE WITHOUT OBSTRUCTION), 531.41(CHRONIC OR UNSPECIFIED GASTRIC ULCER WITH HEMORRHAGE WITH OBSTRUCTION),531.60(CHRONIC OR UNSPECIFIED GASTRIC ULCER WITH HEMORRHAGE AND PERFORATION WITHOUT OBSTRUCTION) ,531.61(CHRONIC OR UNSPECIFIED GASTRIC ULCER WITH HEMORRHAGE AND PERFORATION WITH OBSTRUCTION), 532.00( ACUTE DUODENAL ULCER WITH HEMORRHAGE WITHOUT OBSTRUCTION), 532.01(ACUTE DUODENAL ULCER WITH HEMORRHAGE WITHO OBSTRUCTION), 532.20(ACUTE DUODENAL ULCER WITH HEMORRHAGE AND PERFORATION WITHOUT OBSTRUCTION), 532.21(ACUTE DUODENAL ULCER WITH HEMORRHAGE AND PERFORATION WITH OBSTRUCTION), 532.40(CHRONIC OR UNSPECIFIED DUODENAL ULCER WITH HEMORRHAGE WITHOUT OBSTRUCTION), 532.41(CHRONIC OR UNSPECIFIED DUODENAL ULCER WITH HEMORRHAGE WITH OBSTRUCTION), 532.60(CHRONIC OR UNSPECIFIED DUODENAL ULCER WITH HEMORRHAGE AND PERFORATION WITHOUT OBSTRUCTION), 532.61(CHRONIC OR UNSPECIFIED DUODENAL ULCER WITH HEMORRHAGE AND PERFORATION WITH OBSTRUCTION), 533.00(ACUTE PEPTIC ULCER OF UNSPECIFIED SITE WITH HEMORRHAGE WITHOUT OBSTRUCTION), 533.01(ACUTE PEPTIC ULCER OF UNSPECIFIED SITE WITH HEMORRHAGE WITH OBSTRUCTION), 533.20(ACUTE PEPTIC ULCER OF UNSPECIFIED SITE WITH HEMORRHAGE AND PERFORATION WITHOUT OBSTRUCTION), 533.21(ACUTE PEPTIC ULCER OF UNSPECIFIED SITE WITH HEMORRHAGE AND PERFORATION WITH OBSTRUCTION), 533.40(HRONIC OR UNSPECIFIED PEPTIC ULCER OF UNSPECIFIED SITE WITH HEMORRHAGE WITHOUT OBSTRUCTION), 533.41(HRONIC OR UNSPECIFIED PEPTIC ULCER OF UNSPECIFIED SITE WITH HEMORRHAGE WITH OBSTRUCTION), 533.60(CHRONIC OR UNSPECIFIED PEPTIC ULCER OF UNSPECIFIED SITE WITH HEMORRHAGE AND PERFORATION WITHOUT OBSTRUCTION), 533.61(CHRONIC OR UNSPECIFIED PEPTIC ULCER OF UNSPECIFIED SITE WITH HEMORRHAGE AND PERFORATION WITH OBSTRUCTION), 534.00(ACUTE GASTROJEJUNAL ULCER WITH HEMORRHAGE WITHOUT OBSTRUCTION), 534.01(ACUTE GASTROJEJUNAL ULCER WITH HEMORRHAGE WITH OBSTRUCTION), 534.20(ACUTE GASTROJEJUNAL ULCER WITH HEMORRHAGE AND PERFORATION WITHOUT OBSTRUCTION), 534.21(ACUTE GASTROJEJUNAL ULCER WITH HEMORRHAGE AND PERFORATION WITH OBSTRUCTION), 534.40(CHRONIC OR UNSPECIFIED GASTROJEJUNAL ULCER WITH HEMORRHAGE WITHOUT OBSTRUCTION), 534.41(CHRONIC OR UNSPECIFIED GASTROJEJUNAL ULCER WITH HEMORRHAGE WITH OBSTRUCTION), 534.60(CHRONIC OR UNSPECIFIED GASTROJEJUNAL ULCER WITH HEMORRHAGE AND PERFORATION WITHOUT OBSTRUCTION), 534.61(CHRONIC OR UNSPECIFIED GASTROJEJUNAL ULCER WITH HEMORRHAGE AND PERFORATION WITH OBSTRUCTION), 535.01(ACUTE GASTRITIS WITH HEMORRHAGE), 535.11(ATROPHIC GASTRITIS WITH HEMORRHAGE), 535.21(GASTRIC MUCOSAL HYPERTROPHY WITH HEMORRHAGE), 535.31(ALCOHOLIC GASTRITIS WITH HEMORRHAGE),535.41(OTHER SPECIFIED GASTRITIS WITH HEMORRHAGE), 535.51(UNSPECIFIED GASTRITIS AND GASTRODUODENITIS WITH HEMORRHAGE), 535.61(DUODENITIS WITH HEMORRHAGE), 537.83(ANGIODYSPLASIA OF STOMACH AND DUODENUM WITH HEMORRHAGE), 562.02(DIVERTICULOSIS OF SMALL INTESTINE WITH HEMORRHAGE), 562.03( DIVERTICULITIS OF SMALL INTESTINE WITH HEMORRHAGE), 562.12(DIVERTICULOSIS OF COLON WITH HEMORRHAGE), 562.13(DIVERTICULITIS OF COLON WITH HEMORRHAGE, 569.3(HEMORRHAGE OF RECTUM AND ANUS), 569.85(ANGIODYSPLASIA OF INTESTINE WITH HEMORRHAGE), 578.9(HEMORRHAGE OF GASTROINTESTINAL TRACT UNSPECIFIED), 530.21(ULCER OF ESOPHAGUS WITH BLEEDING), 456.0(ESOPHAGEAL VARICES WITH BLEEDING),456.20(ESOPHAGEAL VARICES IN DISEASES CLASSIFIED ELSEWHERE WITH BLEEDING);
- Hemoptisis - 786.30(HEMOPTYSIS, UNSPECIFIED), 786.39(OTHER HEMOPTYSIS);
- Intra-articular bleeding - 719.10(HEMARTHROSIS SITE UNSPECIFIED), 719.17(HEMARTHROSIS INVOLVING ANKLE AND FOOT), 719.12(HEMARTHORSIS INVOLVING UPPER ARM), 719.13(HEMARTHROSIS INVOLVING FOREARM), 719.14(HEMARTHROSIS INVOLVING HAND),719.15(HEMARTHROSIS INVOLVING PELVIC REGION AND THIGH),719.16(HEMARTHROSIS INVOLVING LOWER LEG),719.19(HEMARTHROSIS INVOLVING MULTIPLE SITES),719.18(HEMARTHROSIS INVOLVING OTHER SPECIFIED SITES),719.11(HERARTHROSIS INVOLVING SHOULDER REGION);
- Upper airway bleeding - 784.8(HEMORRHAGE FROM THROAT);
- Macrohematuria - 599.71(GROSS HEMATURIA);
- Bleeding requiring blood transfusion;
- Surgical procedure for control of bleeding - z3998;
- Fatal bleeding (fatal hospitalization with primary or secondary diagnosis of one of the diagnosis’ defined above).

**Ischaemic events:**

- Acute Myocardial infarction - 410.00(ACUTE MYOCARDIAL INFARCTION OF ANTEROLATERAL WALL EPISODE OF CARE UNSPECIFIED), 410.01(ACUTE MYOCARDIAL INFARCTION OF ANTEROLATERAL WALL INITIAL EPISODE OF CARE), 410.10(ACUTE MYOCARDIAL INFARCTION OF OTHER ANTERIOR WALL EPISODE OF CARE UNSPECIFIED), 410.11(ACUTE MYOCARDIAL INFARCTION OF OTHER ANTERIOR WALL INITIAL EPISODE OF CARE),410.12(ACUTE MYOCARDIAL INFARCTION OF OTHER ANTERIOR WALL SUBSEQUENT EPISODE OF CARE), 410.20(ACUTE MYOCARDIAL INFARCTION OF INFEROLATERAL WALL EPISODE OF CARE UNSPECIFIED), 410.21(ACUTE MYOCARDIAL INFARCTION OF INFEROLATERAL WALL INITIAL EPISODE OF CARE),410.22(ACUTE MYOCARDIAL INFARCTION OF INFEROLATERAL WALL SUBSEQUENT EPISODE OF CARE), 410.30(ACUTE MYOCARDIAL INFARCTION OF INFEROPOSTERIOR WALL EPISODE OF CARE UNSPECIFIED), 410.31(ACUTE MYOCARDIAL INFARCTION OF INFEROPOSTERIOR WALL INITIAL EPISODE OF CARE), 410.32(ACUTE MYOCARDIAL INFARCTION OF INFEROPOSTERIOR WALL SUBSEQUENT EPISODE OF CARE),410.40(ACUTE MYOCARDIAL INFARCTION OF OTHER INFERIOR WALL EPISODE OF CARE UNSPECIFIED), 410.41(ACUTE MYOCARDIAL INFARCTION OF OTHER INFERIOR WALL INITIAL EPISODE OF CARE), 410.50(ACUTE MYOCARDIAL INFARCTION OF OTHER LATERAL WALL EPISODE OF CARE UNSPECIFIED), 410.51(ACUTE MYOCARDIAL INFARCTION OF OTHER LATERAL WALL INITIAL EPISODE OF CARE), ,410.60(TRUE POSTERIOR WALL INFARCTION EPISODE OF CARE UNSPECIFIED),410.61(TRUE POSTERIOR WALL INFARCTION INITIAL EPISODE OF CARE), 410.70(SUBENDOCARDIAL INFARCTION EPISODE OF CARE UNSPECIFIED),410.71(SUBENDOCARDIAL INFARCTION INITIAL EPISODE OF CARE), 410.80(ACUTE MYOCARDIAL INFARCTION OF OTHER SPECIFIED SITES EPISODE OF CARE UNSPECIFIED), 410.81(ACUTE MYOCARDIAL INFARCTION OF OTHER SPECIFIED SITES INITIAL EPISODE OF CARE),410.90(ACUTE MYOCARDIAL INFARCTION OF UNSPECIFIED SITE EPISODE OF CARE UNSPECIFIED), 410.91(ACUTE MYOCARDIAL INFARCTION OF UNSPECIFIED SITE INITIAL EPISODE OF CARE);
- Cerebrovascular accident - 433.01 (OCCLUSION AND STENOSIS OF BASILAR ARTERY WITHOUT CEREBRAL INFARCTION), 433.11(OCCLUSION AND STENOSIS OF CAROTID ARTERY WITH CEREBRAL INFARCTION), 433.01(OCCLUSION AND STENOSIS OF BASILAR ARTERY WITH CEREBRAL INFARCTION), 433.21(OCCLUSION AND STENOSIS OF VERTEBRAL ARTERY WITH CEREBRAL INFARCTION), 433.31(OCCLUSION AND STENOSIS OF MULTIPLE AND BILATERAL PRECEREBRAL ARTERIES WITH CEREBRAL INFARCTION), 433.81(OCCLUSION AND STENOSIS OF OTHER SPECIFIED PRECEREBRAL ARTERY WITH CEREBRAL INFARCTION), 433.91(OCCLUSION AND STENOSIS OF UNSPECIFIED PRECEREBRAL ARTERY WITH CEREBRAL INFARCTION), 434.01(CEREBRAL THROMBOSIS WITH CEREBRAL INFARCTION), 434.11(CEREBRAL EMBOLISM WITH CEREBRAL INFARCTION), 434.91(CEREBRAL ARTERY OCCLUSION UNSPECIFIED WITH CEREBRAL INFARCTION);
- Repeat revascularization procedure (CABG) - z36.11-19
- Repeat revascularization procedure (PCI) - Z0066,z0040 ,z0041,z0042,z0043,z0044,z0045,z0046,z0047,z0048, z3606,z3607,z36060,z36070
